# Correction: Mechanisms of organelle biogenesis govern stochastic fluctuations in organelle abundance

**DOI:** 10.7554/eLife.12522

**Published:** 2015-11-24

**Authors:** Shankar Mukherji, Erin K O'Shea

Mukherji S, O'Shea EK. 2014. Mechanisms of organelle biogenesis govern stochastic fluctuations in organelle abundance. *eLife* **3**:e02678. doi: 10.7554/eLife.02678

Published 10 June 2014

In our originally published manuscript, we erroneously wrote that a Fano factor of 1 marked the boundary between de novo synthesis and fission dominated peroxisome production; the correct Fano factor value for this boundary is 2. We have corrected the text and the location of the green dashed line in Figure 3D, Figure 3—figure supplement 2C, and Figure 4F.

There were four typographical errors in the published manuscript. First, during the process of converting the original submission to the revised submission, an error was introduced in the schematic equation for first order decay. The equation published read:

$$n\overset{\gamma n}{\to }n+1,$$

whereas the correct expression is:

$$n\overset{\gamma n}{\to }n-1.$$

Second, in the x-axis of Figure 1G, the maximum tick label should read ‘10’, not ‘14’.

Third, in the y-axis of Figure 2G, the maximum tick label should read ‘0.4’ rather than ‘0.2’.

Finally, our approximate expression for the Fano factor in the limit where the rate of fusion and first order decay are similar as originally published read:

$$\frac{\sigma^{2}}{\langle n\rangle }=\frac{2k_{de\;novo}+3k_{fission}\langle n\rangle }{3k_{de\;novo}+k_{fission}\langle n\rangle },$$

whereas the correct expression with our approximation scheme is:

$$\frac{\sigma^{2}}{\langle n\rangle }=\frac{2k_{de\;novo}+2k_{fission}\langle n\rangle }{3k_{de\;novo}+k_{fission}\langle n\rangle }.$$

We direct readers to the comment on our paper that provides details of the approximations involved in deriving this expression.

None of these corrections affect the validity of our main conclusions.

The article has now been corrected.

We thank Jeremy Craven for drawing these matters to our attention.

